# Assessment of short-term intraocular pressure parameters in phakic and pseudophakic patients with primary open-angle glaucoma

**DOI:** 10.5935/0004-2749.20210066

**Published:** 2021

**Authors:** Adriana Sobral Lourenço, Candice Cristina Quirino de Araújo, Procópio Miguel dos Santos, Tiago S. Prata, Nara Lidia Vieira Lopes, Regina Cândido Ribeiro dos Santos, Carolina P. B. Gracitelli, Nubia Vanessa Lima de Faria

**Affiliations:** 1 Department of Ophthalmology, Hospital das Forças Armadas, Brasília, DF, Brazil; 2 Department of Ophthalmology, Universidade Federal de Brasilia, Brasília, DF, Brazil; 3 Department of Ophthalmology and Visual Science, Universidade Federal de São Paulo, São Paulo, SP, Brazil; 4 Ophthalmology, Mayo Clinic, Jacksonville, United States; 5 Ophthalmology, Hospital Medicina dos Olhos, Osasco, SP, Brazil; 6 Centro Brasileiro da Visão, Brasilia, DF, Brazil; 7 Ver Mais Oftalmologia, Vinhedo, São Paulo, SP, Brazil; 8 Vera Cruz Oftalmologia, Campinas, São Paulo, SP, Brazil

**Keywords:** Glaucoma, open-angle, Diagnostic techniques, ophthalmological, Drinking water, Intraocular pressure, Glaucoma de ângulo aberto, Técnicas de diagnóstico oftalmológico, Água potável, Pressão intraocular

## Abstract

**Purpose:**

To investigate intraocular pressure peaks in phakic and pseudophakic patients with primary open-angle glaucoma.

**Method:**

Overall, 40 patients with primary open-angle glaucoma were assessed. Of these, 20 patients were phakic and 20 pseudophakic. One eye (randomly selected) was included in the study. All patients underwent the intraocular pressure curves test, followed by the water-drinking test.

**Results:**

A statistically significant difference was observed between the phakic and pseudophakic patients regarding the mean of the peaks in the intraocular pressure curves (p=0.045). A statistically significant intergroup difference was noted regarding the intraocular pressure peaks in the water-drinking test, with higher values observed in the phakic patients (p 0.004).

**Conclusion:**

The intraocular pressure peaks in the water-drinking test and intraocular pressure curves were higher in the phakic group than in the pseudophakic group.

## Abstract (pt)

**Objetivo:**

Investigar picos de pressão intraocular em pacientes fácicos e pseudofácicos com glaucoma primário de ângulo aberto no teste de sobrecarga hídrica.

**Método:**

Quarenta pacientes com glaucoma primário de ângulo aberto foram avaliados; vinte eram fácicos e vinte eram pseudofácicos. Um olho (selecionado aleatoriamente) foi incluído no estudo, todos os pacientes foram submetidos às curvas da pressão intraocular imediatamente após o teste de sobrecarga hídrica.

**Resultados:**

Observou-se uma diferença estatisticamente significante na média dos picos nas curvas da pressão intraocular para os pacientes fácicos e pseudofácicos (p=0,045). Houve diferença estatisticamente significante nos picos da pressão intraocular no teste de sobrecarga hídrica entre os grupos, sendo observados valores mais altos nos pacientes fácicos (p=0,004).

**Conclusão:**

Os picos da pressão intraocular no teste de sobrecarga hídrica foram maiores no grupo fácico que no grupo pseudofácico.

## INTRODUCTION

Glaucoma is a multifactorial optic neuropathy and is the second leading cause of blindness in the world, according to the World Health Organization (WHO) data^(1)^. Even though various risk factors have been attributed to the disease, elevated intraocular pressure (IOP) is considered the primary risk factor^(1)^. Moreover, this is the only risk factor that can be measured and treated. The Ocular Hypertension Treatment Study noted that a 20% reduction in IOP in individuals with ocular hypertension was associated with a greater than 50% reduction in the chance of developing glaucoma^(2)^.

Abnormal IOP is believed to cause anatomic and functional changes in the optic nerve. Therefore, controlling the IOP and ensuring that it is kept stable is still the most effective treatment for glaucoma^(2,3)^. Notably, IOP is known to have a variation of 4-5 mmHg during the day in healthy individuals; however, in patients with glaucoma, this fluctuation might be substantially higher^(4,6)^. Nevertheless, these values could be influenced by other factors, such as the circadian cycle^(7)^, postural position^(8,9)^, and drugs^(10)^. A study by Liu et al.^(4)^ regarding the IOP trends over 24 hours concluded that fluctuations in IOP were greater in patients with glaucoma than in controls. Hence, considering these circumstances, it is essential to study the IOP trends over 24 hours, and the 24-hour curve is one way to understand IOP trend better^(4,11)^.

Nevertheless, other tests to assess short-term IOP va riation have been described, such as diurnal tension curves^(12)^, self-monitoring at home^(13)^, continuous IOP monitoring^(14)^, and provocative tests, such as the waterdrink test (WDT)^(15)^ and IOP measurement with inverted body position (Kanadani test)^(16)^.WDT-first studied by Schmidt in 1928-was initially used to diagnose glaucoma^(17-20)^. Later, the test was almost abandoned because the subsequent studies revealed poor diagnostic performance. More recently, a series of studies suggested that the WDT could assess IOP variation, treatment effectiveness, and disease stability^(21,22)^. Even though studies have revealed a correlation between IOP measurements in the WDT and damage caused by glaucoma, WDT has been used in several studies to estimate the peak IOP, as in this study^(14,23,24)^.

Notably, lens removal is known to cause IOP reduction but does not affect daily IOP fluctuations^(12,25)^. However, it is unknown whether the presence or absence of the lens interferes with the results of the WDT even though this test indirectly measures aqueous humor outflow^(12,25)^.

Therefore, this study investigated and compared the pressure peaks measured in the WDT and outpatient IOP curve (OC) in phakic and pseudophakic patients with primary open-angle glaucoma.

## METHODS

This prospective observational study adhered to the tenets of the Declaration of Helsinki and was approved by the Taguatinga Regional Hospital, Brasília, DF, Brazil. In addition, written informed consent was obtained from all participants.

### Participants

Overall, 40 patients with stable primary open-angle glaucoma were enrolled. Of these, 20 patients were phakic and 20 pseudophakic. All patients were examined by the same physician. They had been using pressure-lowering eye drops (at least one prostaglandin and another class of topical medication) and were not required to stop using any topical eye medication. Patients were recruited from the Hospital Regional de Taguatinga, Distrito Federal, Brazil. The evaluation was performed in the glaucoma sector of the same hospital.

All participants underwent a complete ophthalmological examination, including a review of medical history, best-corrected visual acuity, IOP measurement with Goldmann applanation tonometry (Haag-Streit, Koeniz, Switzerland), slit-lamp biomicroscopy, gonioscopy, refraction, and dilated fundus examination. The criteria for a diagnosis of primary open-angle glaucoma were glaucomatous lesions in the optic nerve, visual field defects and open-angle on gonioscopy with visibility at least as far as the scleral spur. A vertical cup-to-disk ratio of >0.6, asymmetry of the cup-to-disk ratio ≥0.2 between eyes, the presence of nerve fiber layer defects or neuroretinal rim defects like notches, or splinter hemorrhage in the absence of any other pathology that could explain such findings were considered glaucomatous lesions^(14)^. The criteria for visual field defects associated with glaucomatous optic neuropathy were the presence of three non-edge-contiguous test points on the pattern standard deviation plot with p<0.01, with at least one at p<0.005 and a glaucoma hemifield test (GHT) outside normal limits^(14)^.

Patients were excluded if they had had any other ophthalmic surgery (apart from cataract surgery in the preceding 1 year) or laser trabeculoplasty, if they had corneal disorders or eye conditions that could adversely affect IOP measurement, or if they had kidney or heart disease^(14,15)^.

Participants were divided into two groups, one comprising phakic patients with cataracts and the other pseudophakic patients. One eye (randomly selected with the coin flip method) was included in the examination if it had reached the target IOP during routine visits (to avoid bias in terms of glaucoma progression). Pseudophakic patients included in the study had cataract surgery at least 1 year before the study.

### Assessment of WDT and OC parameters

All patients who met the inclusion criteria had their outpatient IOP measured at 8 and 10 am, noon, and 2 and 4 pm, after which they underwent the WDT^(23)^. For the OCs, the highest IOP measured was considered the pressure peak. Similarly, for WDT, the highest IOP measured after the patient drank the water was considered the peak. We decided to use OC because of the practical difficulties associated with the 24-hour curve^(11)^ even though tests based on an OC can fail to detect fluctuations and pressure peaks^(11,26,27)^.

For the WDT, patients drank 1 L of water in 5 minutes and had their IOP measured 15, 30, 45, and 60 minutes later^(28,29)^. They had been instructed not to eat or drink for 2 hours before the WDT^(30)^. The same examiner performed all measurements.

### Statistical analysis

Descriptive analysis was used to present demographic and clinical data. D’Agostino-Pearson’s test was performed to determine whether the data had a normal distribution or not. Descriptive statistics included mean and standard deviation for normally distributed variables and median, quartiles for those distributed non-normally. Wilcoxon test was used to compare the two groups. The computerized analysis was performed using SPSS *“Statistical Package for Social Science for Windows”* (SPSS Inc. version 20). The alpha level (type I error) was set at 0.05.

## RESULTS

Of the 40 patients, 24 were women, and 16 were men. The mean age in the pseudophakic group was 76.00 ± 12.31 years, and in the phakic group was 67.50 ± 6.24 years (p>0.05).

The mean peak pressure measured when taking readings for the OCs was 15.52 ± 1.86 mmHg in the phakic group and 14.15 ± 2.36 mmHg in the pseudophakic group.

The mean baseline IOP was 13.06 ± 2.08 mmHg in the phakic group and 12.2 ± 2.54 mmHg in the pseudophakic group. A statistically significant intergroup difference was noted regarding the mean baseline IOPs (p=0.049).

In the WDT, the mean peak pressure in the phakic group was 18.9 ± 3.8 mmHg and 15.4 ± 3.8 mmHg in the pseudophakic group. Mean IOP measured at 60 min was 15.57 ± 3.45 mmHg in the phakic group and 14.4 ± 3.28 mmHg in the pseudophakic group.

A statistically significant intergroup difference was noted regarding the means of the IOP peaks for both measurement techniques (OC and WDT). The mean of the IOP peaks in the OCs was higher for the phakic patients than for the pseudophakic patients (p=0.045) (Table 1).

**Table 1:** Mean IOP peaks and p values for the outpatient curve and water-drinking test in phakic and pseudophakic patients with glaucoma

	Phakic glaucoma	Pseudophakic glaucoma	p value
MOCP	15.52 ± 1.86 mmHg	14.15 ± 2.36 mmHg	0.045
WDT baseline	13.6 ± 2.08 mmHg	12.2 ± 2.54 mmHg	0.050
WDT peak	18.9 ± 3.8 mmHg	15.4 ± 3.42 mmHg	0.004
WDT 60 min	15.57 ± 3.45 mmHg	14.4 ± 3.28 mmHg	0.273

MOCP= mean outpatient curve pressure; WDT= water-drinking test; Min= minutes; mmHg= millimeter of mercury.

A similar observation was recorded for the means of the IOP peaks in the WDT (p=0.004). However, no statistically significant intergroup difference regarding the IOP measurements at 60 min, although the absolute values were higher in the phakic group than in the pseudophakic group (Table 1).

## DISCUSSION

Glaucoma is an optic neuropathy that can cause blindness if untreated^(1)^. Various risk factors are known to be associated with the pathophysiology of this disease, with IOP being the primary factor and the only one that can be measured and treated^(2)^. Notably, pressure peaks that are not detected during routine outpatient visits are known to be responsible for the progression of glaucomatous damage even in individuals believed to be receiving appropriate treatment^(2-4)^. Therefore, most studies on glaucoma have focused on investigating these pressure peaks. Because a 24-hour curve is incompatible with patients’ daily routines, the OC is used even though it often fails to identify pressure peaks^(11,26,27)^. First described in the 50s, WDT was performed for diagnosis^(31)^. However, the test was later suggested to be used to assess IOP variation, treatment effectiveness, and disease stability^(21,22)^. Nonetheless, when the WDT is compared with other provocative IOP tests (not 24-hour curve), it has been shown to induce intraocular changes higher than what is physiologically expected^(23)^.

In our study, the mean of the peaks observed in the OCs for phakic patients was higher than the corresponding figure for the pseudophakic group. It should be noted that the patients in the latter group had already had phacoemulsification surgery at least 1 year before the study. In a prospective study, Jamil et al.^(25)^ observed a reduction in IOP postoperatively and all follow-up visits. They concluded that phacoemulsification increased the anterior chamber angle width, reducing the number of antiglaucomatous drugs needed to control IOP. Another study by Kim et al.^(12)^ involving 42 eyes of 42 non-glaucomatous patients before and 4 weeks after they had phacoemulsification surgery observed a significant reduction in the mean, maximum, and minimum postoperative IOP. In a retrospective study in 2008, Poley et al.^(32)^ investigated IOP preoperatively and postoperatively in 588 patients with normal IOP or ocular hypertension and noted a reduction in IOP after phacoemulsification, with this reduction being proportional to the preoperative pressure, implying that higher the preoperative IOP, higher would be the postoperative pressure reduction. However, they did not use WDT in their study. Furthermore, in another retrospective study using patient records, Poley et al.^(33)^ observed a statistically significant postoperative reduction in IOP after phacoemulsification compared with preoperative values in patients with primary open-angle glaucoma. Moreover, this reduction was still observed years after the surgery.

Although various studies have reported postoperative IOP reduction after cataract surgery, our review of the literature failed to identify studies regarding the influence of the lens on pressure peaks measured in the WDT. In this study, the IOP peaks in the WDT were higher in the phakic group than in the pseudophakic group. In addition, there was a statistically significant difference between the highest values measured in the OC and WDT.

Moreover, the IOP before and after the WDT was analyzed, and significant differences were observed in both groups, with more differences noted in the phakic group. Notably, previous studies have reported that WDT exhibits a strong correlation with IOP peaks that occur during the day^(21,22)^. However, other studies have revealed that although some WDT and long-term intraocular pressure parameters correlate significantly, WDT might not reflect the long-term IOP profile of patients with stable open-angle glaucoma because of poor agreement^(34)^. Notably, high IOP is the primary risk factor for glaucoma progression and the only modifiable parameter. However, although IOP lowering treatment might decrease disease progression, several patients still experience deterioration. Moreover, various studies have reported that the level of damage measured using the visual field or optical coherence tomography, central cornea thickness, and corneal hysteresis are associated with glaucoma progression^(35,36)^.

Nonetheless, our study had limitations. First, our study sample size was small. Second, the IOP was assessed using OC and not a 24-hour curve. Third, other external factors, such as anterior chamber size or gonioscopy pattern, could influence the results of this present study. Fourth, topical or systemic drugs could affect our results. Moreover, the disease severity and age significantly differed in this study and could affect the final results. Therefore, further studies with a larger sample size should be conducted to confirm these results. However, despite these limitations, this pilot study provides new information that could be confirmed by future studies.

In conclusion, the pressure peaks in the WDT and OC were greater in the phakic group than in the pseudophakic group. None of the studies reviewed had investigated the influence of phacoemulsification on the results of WDT, making this study a pioneering one. Nevertheless, further studies with a larger study population are needed to analyze the preoperative and postoperative pressure peaks in the WDT to determine the influence of phacoemulsification on the results of the WDT. Nonetheless, the results of various provocative tests in pseudophakic patients should be evaluated carefully. Drugs (topical or systemic), circadian cycle, and posture affecting the results of provocative tests or the IOP evaluation^(7-10)^, was an established fact, but the results of this present study reveal that a history of cataract surgery could influence the provocative tests too.
